# Factors influencing screen time in children aged 0–4 years old: a convergent parallel mixed-methods study

**DOI:** 10.1186/s12889-026-28901-0

**Published:** 2026-08-06

**Authors:** Jelle Arts, Teatske M. Altenburg, Sanne L.C. Veldman, Annelinde Lettink, Arnoud P. Verhoeff, Mai J. M. Chinapaw, Jessica S. Gubbels

**Affiliations:** 1 Department of Health Promotion, Maastricht University, NUTRIM Institute of Nutrition and Translational Research in Metabolism, PO Box 616, Maastricht, 6200 MD The Netherlands; 2 Amsterdam UMC location Vrije Universiteit Amsterdam, Public and Occupational Health, De Boelelaan 1117, Amsterdam, The Netherlands; 3 Amsterdam Public Health, Health Behaviors & Chronic Diseases, Amsterdam, The Netherlands; 4 Amsterdam Public Health, Methodology, Amsterdam, The Netherlands; 5 Mulier Institute, Utrecht, The Netherlands; 6 Public Health Service Amsterdam, Sarphati Amsterdam, Amsterdam, 1018 WT The Netherlands; 7 Department of Sociology, University of Amsterdam, Amsterdam, 1018 WV The Netherlands

**Keywords:** Screen use, Determinants, Early childhood, Qualitative research, Longitudinal study

## Abstract

**Background:**

Young children are spending more time on screens at increasingly younger ages, with many exceeding current screen time recommendations. This mixed-methods study aimed to identify factors influencing screen time in children aged 0–4 years.

**Methods:**

As part of the My Little Moves study, we conducted a convergent parallel mixed-methods approach, incorporating a qualitative and quantitative sub-study. In the qualitative sub-study, we examined parental perspectives on which factors influence young children’s screen time through focus group sessions. During these sessions, parents brainstormed on factors they considered to influence young children’s screen time, first individually and thereafter in a group discussion. A thematic analysis using an inductive approach was used to identify categories of parent-perceived factors.

In a separate quantitative sub-study, we collected parent-reported data on parental (e.g., rules around screen time) and environmental factors (e.g., play equipment) through online questionnaires, as well as parent-reported data on children’s screen time through the My Little Moves app, at two timepoints. Robust linear mixed model analyses were used to examine longitudinal associations between factors and screen time.

**Results:**

In the qualitative sub-study, 22 parents listed 46 factors, which were categorized by researchers into: 8 child-related factors, 19 parent-related factors, and 16 environmental factors. The most frequently mentioned factor was screen use as an instrument to distract or occupy the child.

In the quantitative sub-study, complete data were available for 107 children at timepoint 1 (mean age 21.3 ± 12.1 months) and 75 children at timepoint 2 (mean age 28.1 ± 10.6 months). Child age (β = 0.32; 95% CI = (0.22, 0.41)) and the parenting practice of using screen time to control or reward the child’s behavior (β = 0.26; 95% CI = (0.16, 0.37)) were the only factors significantly associated with screen time, both with a moderate effect size.

**Conclusions:**

Findings from this study suggest that parental use of screen time to distract, reward, or control children’s behavior contributes to increased screen time in 0–4-year-old children. These practices could be targeted to reduce screen time in early childhood.

## Background

The widespread availability of screens, such as TVs, tablets and smartphones, has contributed to a marked increase in screen time in early childhood (0–5 years) [1]. Over the past decade, children under the age of five have become the fastest-growing group of users of screen media [2]. Limiting screen time in early childhood is important due to the potential negative impact of screen time on young children’s healthy development [3]. Screen time in early childhood has been associated with delays in language development, impaired executive functioning, and poorer psychosocial well-being [4–6]. Additionally, high screen time has been associated with poorer sleep, less physical activity, and increased overweight in early childhood [7–10]. Furthermore, studies suggests that screen time in early childhood tracks into later childhood, reinforcing unhealthy patterns of screen use and affecting overall health and development in the long run [4].

In addition to the amount of screen time, the content, type, and context of screen use may also influence children’s developmental outcomes. For example, evidence suggests that slow-paced, educational content may have less adverse, or even beneficial, effects compared with fast-paced, entertainment-oriented content, particularly with respect to language development, executive functioning, and socio-emotional development [11–13]. Furthermore, co-viewing with parents and active interaction during screen use may enhance potential learning benefits and mitigate some adverse effects [11, 13]. However, evidence regarding the influence of screen content, type, and context during early childhood remains limited and heterogeneous [11, 14]. As such, reducing overall screen time remains an important public health target.

Therefore, the World Health Organization has established guidelines on screen time for children under 5 years of age [15]. These guidelines recommend that children under 2 years of age should not have any screen time, while children aged 2 to 5 years should have no more than one hour of screen time per day. Unfortunately, a significant proportion of young children exceed the suggested screen time limits: a recent meta-analysis, including mostly studies from high-income countries, found that only 24.7% of children younger than 2 years, and 35.6% of children aged 2 to 5 years met the screen time recommendations [1].

Although some interventions have been able to induce significant reductions in screen time in early childhood, these have mostly focused on children aged 3 to 5 years and were largely implemented within childcare or preschool settings [16]. However, previous research suggests that interventions may be more beneficial when aimed at even younger children, highlighting a gap in evidence for interventions aimed at children under the age of 3 years, particularly in the home-based setting [16]. In this setting, it is important to recognize that the home environment presents unique challenges and constraints, as screen use in early childhood is shaped not only by parenting practices but also by broader family circumstances and everyday practical demands, such as limited access to childcare, care for multiple children or older siblings, and time pressures related to work and household responsibilities.

To develop targeted and effective interventions, it is essential to understand factors that influence screen time in early childhood [17]. A systematic review on factors associated with screen time in children aged 0–5 years found evidence for several factors, particularly in the physical environmental domain (e.g., the presence of electronic devices in the bedroom) and the social/cultural domain (e.g., parental self-efficacy in limiting children’s screen time, parental screen time) [17]. However, for most factors the evidence was inconsistent or insufficient [17]. Additionally, most studies were cross-sectional, whereas longitudinal studies provide stronger evidence for causality [17]. Moreover, there is a need for studies to explore moderating factors, e.g. some factors may be more or less relevant for screen time depending on the child’s age [18, 19].

While quantitative studies provide valuable insights into associations between specific factors and screen time, some important factors may have been overlooked or are difficult to measure [17]. Therefore, qualitative research serves as a valuable complementary approach, offering deeper insights into factors shaping screen time among young children [20]. Examining a broad range of factors in focus groups with parents, Decker et al. [21] found that weather conditions and parental screen-viewing habits at home were the most important factors influencing screen time among children aged 4 to 6 years [21]. Few qualitative studies have explored the factors influencing screen time in children under 4 years of age, and those that have primarily focused on parental factors [22–24]. Identified factors include parental attitudes towards screen use and parenting practices such as using screens as educational tools or digital babysitters [22]. In children under the age of 2 years, Golden et al. [25] investigated parental decision-making around infants’ smartphone use, while Hesketh et al. [19] explored parents’ perspectives on their role in influencing screen time behaviors in children under 12 months and those aged 3–5 years [19, 25]. Additionally, Mallawaarachchi et al. [24] examined parental motives for providing smartphones and tablets to children aged 1–6 years [24]. Moreover, Carson et al. [26] investigated parental perspectives on the Canadian sedentary behavior guidelines for 0–4-year-olds, including perceived barriers in meeting the screen time recommendations [26]. While these studies provide valuable insights into parental factors, they do not fully capture the broader socioecological context that may influence screen time in early childhood, including child-related, environmental, and societal influences, such as limited opportunities for play, work–family structures, and the increasing digitalization of everyday life. There remains a lack of comprehensive qualitative research that considers individual, parental, and environmental factors that potentially influence screen time in the entire age range of 0–4 years.

The present study investigated individual, parental and environmental factors that potentially influence screen time in young children, using a mixed-methods approach. Through a qualitative sub-study, we examined parents’ perspectives on which factors influence screen time in children aged 0–4 years. Through a quantitative sub-study, we additionally examined longitudinal associations between potential factors and screen time in children aged 0–4 years, as well as potential interactions with the children’s age.

## Methods

**Fig. 1:**
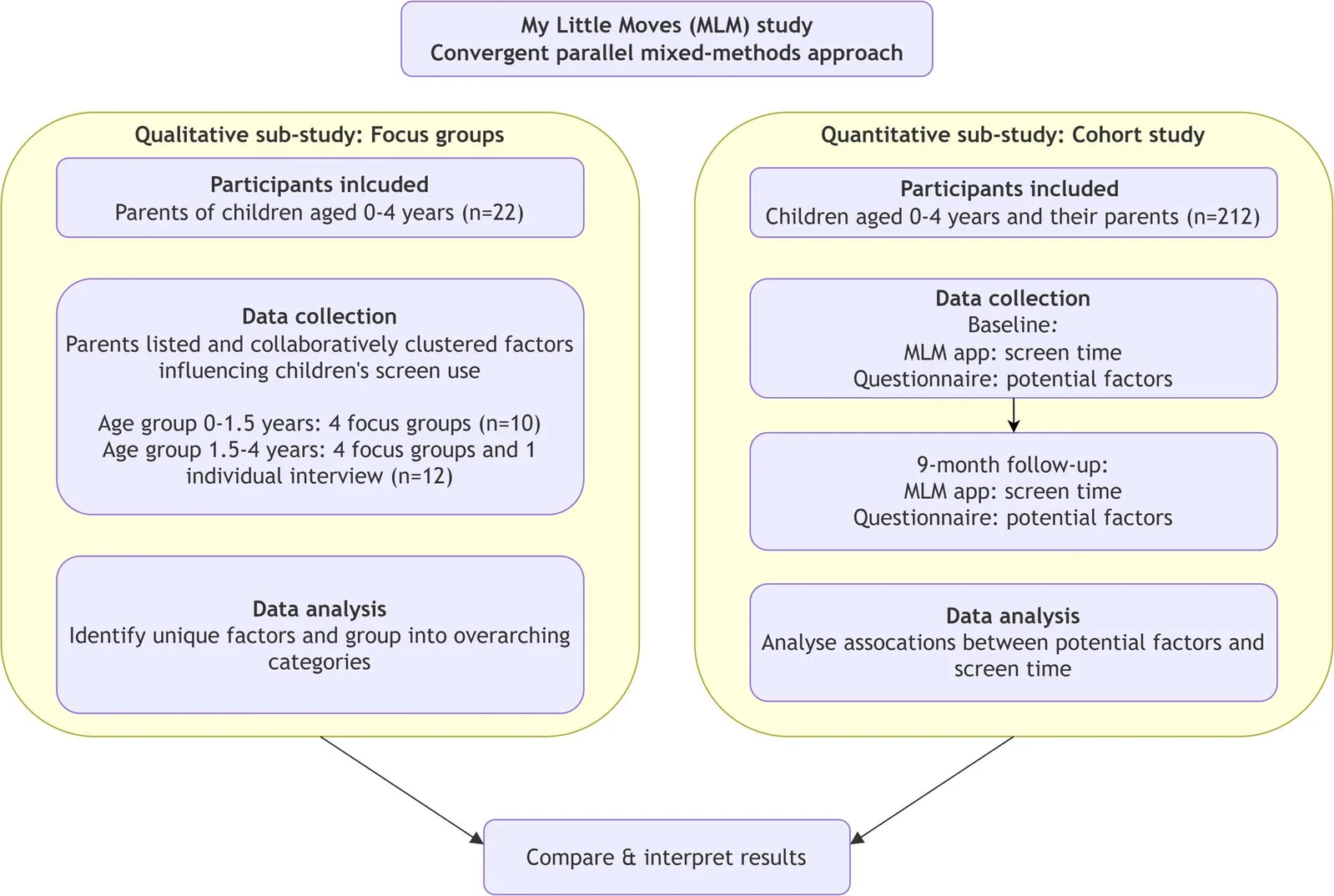
Overview of the convergent parallel mixed-methods approach within the My Little Moves (MLM) study, including the qualitative and quantitative sub-study. Note: more detailed participant numbers of the quantitative sub-study are presented in Fig. 2

The current study is part of My Little Moves, a study conducted in the Netherlands that aimed to 1) develop measurement instruments to assess young children’s 24-hour movement behaviors; [2] examine the prospective association between 24-hour movement behaviors and young children’s growth, motor and social-emotional development; and [3] examine factors influencing young children’s 24-hour movement behaviors, including screen time [27]. To investigate factors potentially influencing young children’s screentime (aim 3), the present study used a convergent parallel mixed-methods approach, incorporating a qualitative and quantitative sub-study [28]. In this approach, data from each method are collected simultaneously and analyzed separately before being compared and interpreted [28]. Figure 1 presents an overview of our convergent parallel mixed-methods approach, including both sub-studies. The qualitative sub-study involved online focus group sessions with parents of children aged 0–4 years, aiming to identify parents’ perspectives on factors influencing young children’s screen time. The quantitative sub-study was based on data from the My Little Moves cohort study in which data were collected on factors potentially influencing screen time in children aged 0–4 years. Data from two timepoints (baseline and nine months later) were used to examine longitudinal associations between these factors and young children’s screen time. Data on young children’s screen time were collected by parent-report using the My Little Moves app [29, 30]. Data on factors potentially influencing screen time were collected online using the My Little Moves determinants questionnaire, which included items adapted from previously developed and validated questionnaires [27]. A detailed description of the My Little Moves cohort study is available elsewhere [27].

### Qualitative sub-study: focus groups

#### Participants and recruitment

Due to the rapid growth and development during early childhood, we conducted separate sessions for parents of children aged 0–1.5 years (up to 18 months) and those aged 1.5–4 years (18 to 48 months). This cut-off was chosen as most typically developing children reach the motor milestone of walking before or around the age of 18 months [31], which reflects increased mobility and environmental exploration that may substantially alter the factors influencing children’s screen use. We did not screen children’s development (e.g., whether children reached the motor milestone walking or not). Each session included three to six participants. Although in-person focus groups typically consist of six to eight participants, smaller groups were chosen to accommodate better discussions in an online setting [32].

Parents were eligible for inclusion if they had a child aged 0–4 years and were proficient in the Dutch language. Exclusion criteria included parents of children born extremely preterm (< 32 weeks) and parents of children with developmental disorders or medical diagnoses, as reported by the parents. We aimed to include at least 20 parents, with at least 10 participants in each age group. The study protocol was approved by the Medical Ethics Committee of the VU University Medical Center (METC VUmc; No. 2021.0660).

Parents were recruited through a variety of settings, including early childhood education and care (ECEC) services (e.g., preschools) and sport clubs. Participating organizations approached parents by distributing information letters via email or handing out flyers. Furthermore, the research team utilized personal networks and snowball sampling techniques to recruit additional parents as part of a convenience sample. Informed consent was obtained from all parents prior to their participation in the study, using the survey software ‘Survalyzer’ (Survalyzer BV, Utrecht, the Netherlands).

#### Procedures

Data were collected between April and May 2023. Parents provided their demographic background characteristics through an online survey incorporated in the informed consent form, including their country of birth, age, gender, highest level of education (categorized as lower, medium, and higher according to the International Standard Classification of Education [33]), the number of children in their household and the age of these children. Next, a focus group session was scheduled with the parents, involving one parent per child. If parents were unable to attend a group session, efforts were made to arrange an individual session. The sessions were conducted through secured online video-meetings using the software ‘Zoom’ [34]. Two trained research assistants (AP and HF) facilitated the sessions: one served as the moderator, while the other took notes, provided technical support, and assisted in moderating the session when necessary. Both research assistants were supervised by experienced researchers (JA, TA, and JG) with expertise in qualitative research methods [30, 35–38]. Each session lasted approximately 1 to 1.5 h and was recorded using a voice recorder (Olympus VN-5500PC).

#### Data collection

A step-wise approached was developed for the focus groups, based on the concept mapping method [37–39]. The sessions consisted of four steps:

- Step 1 (± 10 min): Each focus group session began with participant introductions, followed by a brief presentation outlining the study’s objectives and an overview of the session’s activities.
- Step 2 (± 10 min): Parents were asked to individually brainstorm and list as many factors as possible by answering the question: *“What determines whether or not your child*,* or a child of a similar age*,* uses a screen?“.* If needed, additional clarification was provided by the facilitators to ensure this question was interpreted consistently by all participants. Using the web-based whiteboard tool ‘Google Jamboard’, parents individually reported all listed factors.
- Step 3 (± 20 min): Parents shared their listed factors within the group and had the opportunity to clarify their input. They were also encouraged to add new factors when inspired by others’ contributions or shared experiences. New factors were either added by parents directly in Google Jamboard or noted by the facilitator. The facilitator compiled all listed factors into a single Word document.
- Step 4 (± 20 min): Parents collaboratively grouped the listed factors into clusters, based on similarity. To facilitate this process, the moderator shared the Word document displaying all the factors. Each parent was given a few minutes to individually think about how the factors might be grouped and what could be appropriate names for the clusters. Parents then shared their ideas, discussed them as a group, and reached consensus on the best way to cluster the factors and the name of each overarching cluster.

In case of an individual session, the participating parent was guided through similar steps by the facilitator but there was no discussion with other parents. Sessions were organized until data saturation was reached. Data saturation occurred when no new factors emerged during the sessions.

#### Data analysis

Each focus group or individual session resulted in an overview of clusters of parent-perceived factors influencing children’s screen time, either for 0–1.5-year-old or 1.5–4-year-old children. A thematic analysis was conducted using an inductive approach [29] to develop one comprehensive overview of categories of parent-perceived factors influencing young children’s screen time for both age groups. This analysis was done by two researchers (JA and a trained research assistant, AP) through the following three steps:

- Step 1: All collected data, including clusters of factors from each session, factor descriptions, and session notes, were read multiple times to get a thorough understanding of the data.
- Step 2: Parent-perceived factors from all sessions were combined in one list of factors. Factors that were described differently by parents across sessions but had a similar meaning were combined. As a result, one list with unique factors was created.
- Step 3: All unique factors were grouped into overarching categories and sub-categories through axial coding. As much as possible, we adhered to clusters created by parents during the focus group sessions. However, due to the wide variation in the naming and content of clusters created across different focus groups, the created sub-categories were largely based on the socioecological model previously used by Sallis et al. [40]. In case of doubts or discrepancies, a third researcher (JG) was consulted to reach consensus.

### Quantitative sub-study: cohort study

#### Participants and recruitment

Children were included in the quantitative study if they were (1) aged 0–4 years (up to 48 months), (2) born not extremely premature (< 32 weeks), and (3) without parent-reported developmental disorders or medical diagnoses that might influence the 24-hour movement behaviors or the development of the child. Additionally, parents needed to be able to read the Dutch language and own a smartphone or tablet device. The Medical Ethics Committees of the VU University Medical has approved the study protocol (No. 2022.0020).

Children were recruited via their parents through the dynamic Sarphati Cohort, which includes children aged between 0 and 4 years in Amsterdam [41]. Parent-child dyads participating in the Sarphati cohort received an information letter about the My Little Moves study from Sarphati through email. In addition, we recruited parents and their children through ECEC services, community centres, sports clubs, or public spaces such as playgrounds. Here, parents received an information letter through email, newsletters and/or flyers. Informed consent was obtained from all parents before participating in the study, using the survey software ‘Survalyzer’.

#### Procedures

Data for timepoint 1 were collected between June 2022 and August 2023, and for timepoint 2 between March 2023 and April 2024. Parents provided their child’s date of birth and sex through an online survey incorporated in the informed consent form. Subsequently, parents received an email in which they were asked to complete the My Little Moves app for seven consecutive days. They also received detailed information on how to download and complete the My Little Moves app. In addition, parents received an email through Castor EDC with a link to an online questionnaire on potential factors influencing their child’s screen time and parental characteristics. Parents who did not yet report any of their child’s activities in the My Little Moves app and/or complete the online questionnaire received reminders after one and three weeks.

#### Screen time – my little moves app

Children’s screen time was assessed using the My Little Moves app. The app has a time-use diary format thereby enabling parents to report their child’s activities throughout the day, among which was screen use [29, 30]. Before completing the time-use diary, parents provided their child’s age (0–6 months, 6–12 months, 1–2 years, 2–3 years, and 3–4 years) and achieved motor milestones (e.g., whether child is able to sit without support, crawl, and/or walk without support) in the app. This information enabled the app to tailor its content to the child’s developmental stage. For the activity category screen use, parents were prompted to provide additional details on the intensity of movement during screen use (active play/calm play/I don’t know), device type (television/tablet/smartphone/game console or computer/other/I don’t know), and other people present (one or more other children/one or more other adults/both other children and adults/my child was alone/other/I don’t know). In a content validity study, the My Little Moves app was found to be sufficiently relevant, comprehensive, comprehensible and user-friendly [30]. Completing the app took parents between 10 and 30 min per day.

In a reliability and validity study, based on a comparative analysis with accelerometer data, the My Little Moves app’s ability to assess young children’s movement behaviors was confirmed [29]. App data were considered reliable when parents completed the app for at least two days with ≥ 23 h of activities, excluding the categories ‘other activity’, ‘child was with someone else’, and ‘I don’t know’ [29]. For each participant that met these criteria for reliable data, screen time was calculated by summing activities reported as “screen use” for each day, and averaging these daily totals.

#### Parental and physical environmental factors

Based on previous literature and expertise of our research team, we collected data on five parental factors and three physical environmental factors that we hypothesized to be associated with young children’s screen time. One of the environmental factors included was temperature. Daily outdoor temperature data were obtained from the Royal Netherlands Meteorological Institute (KNMI), recorded at a central location in the Netherlands (i.e., De Bilt) [42]. The maximum temperature on specific days was matched with the dates on which participants completed the My Little Moves app. Subsequently, the average maximum temperature was calculated across all completed days.

All other factors were assessed using a questionnaire based on items from the Parenting SOS questionnaire [43, 44], the SUNRISE Study Parent Questionnaire [45], and the Environment and Policy Assessment and Observation (EPAO) instrument [46, 47]. The physical environmental factors included: (1) presence of screens in the living room (one item), and (2) availability of active indoor play equipment (11 items). For the latter, a sum score was calculated based on the number of items of equipment present (range: 0–11). The parental factors included: (1) promoting screen time for education (one item), (2) promoting screen time to control or reward behavior (four items, Cronbach’s α = 0.62 in our sample), (3) role modelling of screen time (one item), (4) rules around screen time (two items, Cronbach’s α = 0.84 in our sample), and (5) monitoring of screen time (one item). For promoting screen time to control or reward behavior, a mean score of the four items was calculated (range: 0–5). For rules around screen time, a sum score of the two items was calculated (range: 0–2). Table 1 provides an overview of these factors, including the questionnaire items, response options and source. Compared with the original questionnaires, several items were slightly rephrased based on consensus within our research team to improve consistency in wording across the questionnaire (e.g., using “screen” instead of “electronic screen device”) or to improve clarity for participating parents (e.g., “Walking car, balance bike, tricycle or other toys to ride on” instead of “Riding toys (tricycles, cars, etc.)”).

**Table 1:** Overview of factors assessed through parent-report, including the questionnaire items, and response options, and source

Factor	Questionnaire items	Response options	Source
Parental			
Promoting screen time for education	How often do you use a screen to educate your child?	6-point Likert scale ranging from ‘Never’ to ‘Multiple times per day’	Translated and adapted from Parent/Caregiver questionnaire SUNRISE [45] No validity/reliability information
Promoting screen time to control or reward behavior	1. How often do you use a screen to calm down your child when he/she is upset? 2. How often do you use a screen to keep your child busy while you do something else? 3. How often do you offer screens to your child as a reward for good behavior? 4. How often do you take away screens to your child as a punishment for bad behavior?	6-point Likert scale ranging from ‘Never’ to ‘Multiple times per day’	Translated and adapted from Parenting SOS study: Screen time parenting, subscale ‘use of screen time to reward/control child behavior’ [43, 44] Internal consistency scores from original subscale [44]: Cronbach’s α = 0.79 Factor loadings = 0.57–0.94
Role modelling of screen time	How often do you use screens with your child present?	6-point Likert scale ranging from ‘Never’ to ‘Multiple times per day’	Translated and adapted from Parent/Caregiver questionnaire SUNRISE [45] No validity/reliability information
Rules around screen time	1. Do you have rules regarding how much time your child is allowed to use screens? 2. Do you have rules regarding when your child is allowed to use screens?	Dichotomous (Yes/No)	Added No validity/reliability information
Monitoring of screen time	Do you monitor the screen use of your child?	Dichotomous (Yes/No)	Translated and adapted from Parenting SOS study: Screen time parenting, subscale ‘Limiting/monitoring of screen time’ [43] No validity/reliability information for single item
Physical environmental			
Presence of screens in the living room	Are there screens in the living room?	Dichotomous (Yes/No)	Added
Availability of active indoor play equipment	Which of the following play equipment do you have available for your child inside your home?	(1) Ball or other toy to throw or roll; (2) Football goal, basketball hoop, etc.; (3) Loose toys for jumping, e.g., skippy ball; (4) Play mat or other floor covering for playing on the floor; (5) Walking car, balance bike, tricycle or other toys to ride on; (6) Rocking horse or other materials to bounce on; (7) Sandpit and/or water toys (e.g., shovels, buckets); 8) Climbing equipment (e.g., climbing frame or slide); 9) Swing; 10) Possibility to play music or musical instruments; 11) Other play equipment	Adapted from the Dutch version of EPAO [47] Test-retest reliability and validity (gold standard observer) scores of the original subscales [46]: Physical activity environment Portable equipment subscale: ICC = 0.93-0.96 (observer) ICC = 0.59-0.74 (staff) *r* = .23-0.26 Inter-rater reliability and validity (compared to accelerometer) scores of the original subscales [48]: Indoor play equipment subscale: ICC = 0.81 *r* = .11

*Abbreviations*: *EPAO* Environment and Policy Assessment and Observation, *ICC* Intraclass Correlation Coefficient, *r* correlation coefficient

#### Participant characteristics

Parents reported the country of birth of both the mother and father (categorized as the Netherlands or other), their date of birth, gender (male, female, or other), and highest level of education (categorized as lower, medium, or higher according to the International Standard Classification of Education [20]). The age of participating parents was calculated based on their date of birth and the date of questionnaire completion. Similarly, the child’s age was determined using their date of birth.

#### Analysis

Statistical analyses were conducted using RStudio version 2024.12.0. Statistical significance was set at alpha = 0.05. As screen time was not normally distributed, median screen time and interquartile ranges (IQRs) were calculated. Additionally, we calculated percentages for all follow-up questions in the MLM app: screen time intensity, the type of screen used, and the people present. For all potential factors and participant characteristics, medians and IQRs or means and standard deviations were calculated for numeric variables, while percentages were calculated for categorical variables.

We assessed the assumptions for linear mixed model analysis through visual inspection of residual plots and found that the normality assumption was violated. Despite multiple transformations, the residuals continued to deviate from a normality. Therefore, we used robust mixed models to analyse longitudinal associations, employing the rlmer function from the robustlmm package in R [49]. These models included all factors, child sex and age as fixed effects, and a random intercept for participant id to account for repeated measurements. Participants were included if they had complete data on screen time and potential factors at least at one time point. A sensitivity analysis was conducted to compare associations among participants with complete data at both timepoints against the main analysis. Standardized beta coefficients were calculated to assess effect sizes, which were interpreted as weak (< 0.2), moderate (0.2–0.5), and strong (> 0.5) [50].

Since we hypothesized the child’s age to influence the association between factors and screen time, we additionally examined interaction effects between age and each factor. We conducted separate robust mixed models for each factor, incorporating all factors, the child’s sex and age, and the interaction between age and the specific factor as fixed effects, while including a random intercept for participant id to account for repeated measurements. Significant interaction effects were further explored through visual inspection of interaction plots and stratified analyses by age group.

## Results

### Qualitative sub-study: focus groups

#### Participant characteristics

Twenty-two parents participated in this sub-study, with two to six parents per focus group session, and one parent participating in an individual session. Four focus groups with in total 10 parents focused on 0–1.5-year-old children, and four focus groups with in total 11 parents and one individual session focused on 1.5–4-year-old children. Table 2 presents the demographic characteristics of participating parents. Most parents identified as female (18 out 22), and were highly educated (19 out of 22), with ages ranging from 23 to 42 years.

**Table 2:** Demographic characteristics of participating parents in the qualitative sub-study (*n* = 22)

Characteristic	Age group: 0–1.5 years	Age group: 1.5–4 years
Gender (female / male)	9 / 1	9 / 3
Age in years (mean ± SD, range)	33.3 ± 2.6 (30–38)	35.0 ± 5.4 (23–42)
Educational level (lower / medium / higher)	0 / 1 / 9	2 / 0 / 10
Country of birth (the Netherlands / other)	9 / 1	11 / 1
No. of children (mean ± SD, range)	1.8 ± 0.6 (1–3)	1.4 ± 0.5 (1–2)
Age children (0–6 months / 6–12 months / 1–2 years / 2–3 years / 3–4 years / older)	4 / 4 / 3 / 5 / 2 / 0	1 / 1 / 1 / 8 / 2 / 4

#### Parent-perceived factors

Table 3 presents all parent-perceived factors influencing screen time, and the number of sessions in which factors were indicated. All factors were grouped into three overarching categories, namely child factors, parental factors and environmental factors. For each category, the factors that were indicated in at least two sessions are described below. If a factor is described without specifying an age group, it applies to both 0–1.5-year-old and 1.5–4-year-old children.

**Table 3:** Parent-perceived factors influencing screen time, grouped in child, parental, and environmental factors

Child factors	Parental factors	Environmental factors
Demographic and biological	Biological	Physical environmental
Illness + (4)	Illness + (1) I	Bad weather conditions + (4)
Age + (1) T		Presence of screens + (3)
	Behavioral attributes and skills	Availability of play equipment – (2)
Behavioral attributes and skills	Negative attitude towards screen time/motivation to limit screen time – (5)	Availability of organized age-appropriate activities – (1) I
Interest in screens + (4)	Parental screen time + (3)	Attractive design of screens + (1) I
Addiction to screens + (4)	Placing high importance and value on physical activity – (3)	Social environmental
Ability to entertain themselves without screens – (2) T	Willingness/ability to keep child occupied with activities without screens – (3)	Older siblings + (4)
Knowledge of how to use screens + (1) T	Awareness of the effects of screen time – (2)	Connection with family + (4)
Cognitive, emotional, and psychological		Informal childcare +/- (2)
Moodiness + (2) T		Number of children in the household + (2) T
Tiredness + (3) T		Number of children in the neighborhood – (1) T
	Cognitive, emotional, and psychological	Proximity of friends and family – (1) I
	Tiredness + (2)	Sibling plays with the child – (1) I
		Peers/friends are using screens + (1) I
	Parenting practices	Policy
	Screen to distract child/keep child occupied + (9)	Policy regarding screen use at ECEC services +/- (4)
	Screen to educate child + (7)	ECEC subsidy – (1) I
	Screen to calm/comfort child + (5)	Availability of parental leave – (1) I
	Screen to dance/listen to music + (5)	
	Rules around screen time – (5)	
	Screen to reward behavior + (3)	
	Screen time during eating + (3)	
	Screen to entertain child + (3) T	
	Monitoring of screen time – (1) T	
	Screen time in daily routine + (1) T	
	Take away screen to punish behavior + (1) T	
	Parent-child co-watching + (1) T	

Factors perceived as increasing screen time are indicated with +, while factors decreasing screen time are indicated with –; The number of sessions in which each factor was mentioned is indicated between brackets (). Nine sessions were conducted, of which four focused on 0–1.5-year-old children, and five on 1.5–4-year-old children; I indicates factors that were only mentioned for infants (0–1.5-year-old children); T indicates factors that were only mentioned for toddlers (1.5–4-year-old children)

##### Child factors

Parents identified a total of eight child factors, that were grouped into three sub-categories: two demographic and biological factors, four factors related to behavioral attributes and skills, and two cognitive, emotional and psychological factors. Six factors were indicated in at least two sessions.

Demographic and biological factors.

Across both age groups, parents indicated that illness of the child was indicated as a reason for excessive screen time across both age groups. As one parent stated (age category: 0–1.5 years):

The exception at our house is if they are ill; then they are allowed to watch everything, unlimited.

Behavioral attributes and skills.

Parents noted that screen time increased when children developed a stronger interest in screen time, or even an addiction to screen time. One parent described (age category: 1.5–4 years):

As she gets older, she starts asking about it more often, so the child becomes addicted and wants to watch more. There is an attraction to screens, it’s unbelievable.

Moreover, for children aged 1.5–4 years, parents observed differences between children in their ability to entertain themselves without screens.

Cognitive, emotional, and psychological.

For children aged 1.5–4 years, parents indicated that children’s desire for screen time tends to increase when they are tired. One parent explained (age category: 1.5–4 years):

If my child has finished playing [off-screen] and is tired or has no energy, especially at the end of the day, then a screen is used.

Similarly, parents of children in this age group indicated that they are more likely to allow screen time when their child is moody.

##### Parental factors

Parents identified nineteen parental factors that were grouped into four sub-categories: one biological factor, five factors related to behavioral attributes and skills, one cognitive, emotional, and psychological factor, and 12 parenting practices. Fourteen factors were indicated in at least two sessions.

Behavioral attributes and skills.

Parental attitudes toward screen use were mentioned as a factor, with parents expressing negative views about screen time for young children. One parent explained (age category: 0–1.5 years):

I think they are way too young to put them behind screens, especially because they can play in the playpen if I have to do something else in the house.

Attitudes toward screen use or the motivation to limit it can differ between parents. For example, one mother shared (age category: 1.5–4 years):

I do notice that when she is with her father, she uses more screens. She often asks if she can watch something, and then her father allows it. I think because he does not think it is harmful for the children to watch something on the iPad for example.

Parental awareness of the effects of screen time was also indicated as a factor. Some parents had been advised by healthcare professionals to reduce screen exposure to improve their child’s sleep or vision, while others viewed excessive screen time as detrimental to children’s development. One parent shared (age category: 1.5–4 years):

I also think it is important that she participates in the conversation when we are sitting at the table, and if you put an iPad in between, I am sure that conversation will not take place.

Additionally, parents noted that the value they place on physical activity and other off-screen activities, as well as their willingness and ability to support their children in engaging in these activities, influences children’s screen time. For example, going to play outside or reading together emerged as important off-screen activities. One parent noted (age category: 0–1.5 years):

If he is playing inside or outside, then [he gets] no screens in any way because I think it is important that my son can enjoy time by himself when he is playing.

Moreover, parents indicated that their own screen time influenced their children’s screen habits. As one parent observed (age category: 1.5–4 years):

If parents watch a lot of TV themselves, it is also something that is easily adopted, and then it becomes more normal.

Cognitive, emotional, and psychological.

Parents reported being more likely to allow screen time when they themselves felt tired. As one parent noted (age category: 0–1.5 years):

When a parent has no energy left, a screen is often used as a moment of rest.

Parenting practices.

Several parenting practices were identified by parents as influential, with the use of screen time as an instrument to distract children or to keep children occupied being mentioned in every session across both age groups. Many parents use screens to keep children occupied while managing other tasks, such as household chores or caring for siblings, or simply to have some private time. For example, one parent shared (age category: 1.5–4 years):

When I am home with the kids, and busy working, I am sometimes tempted in the morning to put them behind a screen so I can update my email. And if I have to do things at home, then it is sometimes nice to use screens because then they [the children] do not need me.

Similarly, another parent noted (age category: 0–1.5 years):

In the car and on vacation, she can watch videos more often, especially when we’re at a restaurant where she has to sit still and is already tired, so she needs something to keep her entertained.

Additionally, parents noted that screens can be used to redirect a child’s attention away from their current activity. For example, screen time was sometimes used during meals to encourage eating or to help keep the child seated while potty training. Furthermore, parents indicated that setting rules helps limit screen time for children in both age groups. For example, they restricted screen use on weekdays or before bedtime. Screens were also used by parents to calm or comfort children, for example as a moment of rest or when children are upset. Moreover, screens were used as a reward for good behavior, such as when finishing eating their food. Another reason for screen use was education. For example, parents reported allowing their children to watch programs to support language development. Similarly, screens were used for dancing or listening to music. Entertainment as the primary reason for screen time, such as using screens when children are bored, was mentioned only for the 1.5–4-year age group.

##### Environmental factors

Parents identified 16 environmental factors that were grouped into three sub-categories: five physical environmental factors, eight social environmental factors, and three policy related factors. Seven factors were indicated in at least two sessions.

Physical environmental.

Parents reported that bad weather and the presence of screens contribute to increased screen time in young children. They noted that screens are deeply integrated into daily life in modern society, which makes screen exposure almost unavoidable. However, parents also indicated that when more off-screen play equipment is available, children are more likely to engage in alternative play activities, which can help reduce their screen time.

Social environmental.

Having older siblings was mentioned as influencing young children’s screen time. Parents noted that it can become more challenging to manage the younger children’s screen time when older siblings are allowed to use a screen more frequently. For example, one parent shared (age category: 1.5–4 years):

I have an older child of 6, and that is really an essential difference [between both children]. It was very easy to determine what they watch and how long when the eldest was younger, but now we have an age difference, and that is a clear factor. One is 6, and the other is 2.5 years, so you can no longer let the younger one not watch if the older one does. Now, the youngest automatically has more screen time than is desired.

In line with this finding, parents of children aged 1.5 to 4 years identified having more children in the household as a factor contributing to increased screen time. For example, one parent noted (age category: 1.5–4 years):

*“The size of the family plays a major role. From what I observe*,* in larger families—particularly when the children are close in age—parents often turn to screens as a way to maintain a calm household.”*

Additionally, it was indicated that screens are used for digital communication, such as FaceTime, to connect with family members. Informal childcare, such as care provided by grandparents or babysitters, was also identified as a factor influencing children’s screen time in both directions. For example, some parents noted that their children often had more screen time when staying with their grandparents, while others mentioned that grandparents strictly limited screen time.

Policy.

The screen time policy at ECEC services was a mentioned factor for both age groups. Most parents indicated that the ECEC service their child attended allowed little to no screen time. However, there were some exceptions, as one parent explained (age category: 0–1.5 years):

*“There are also childcare centers where they watch a lot of television as a reward. When the children have played outside*,* they can watch videos afterward. But I know from my childcare center that they only put on a video just before lunch*,* and for the rest of the day*,* they do not use any type of screen.”*

### Quantitative sub-study: cohort study

#### Descriptives

**Fig. 2:**
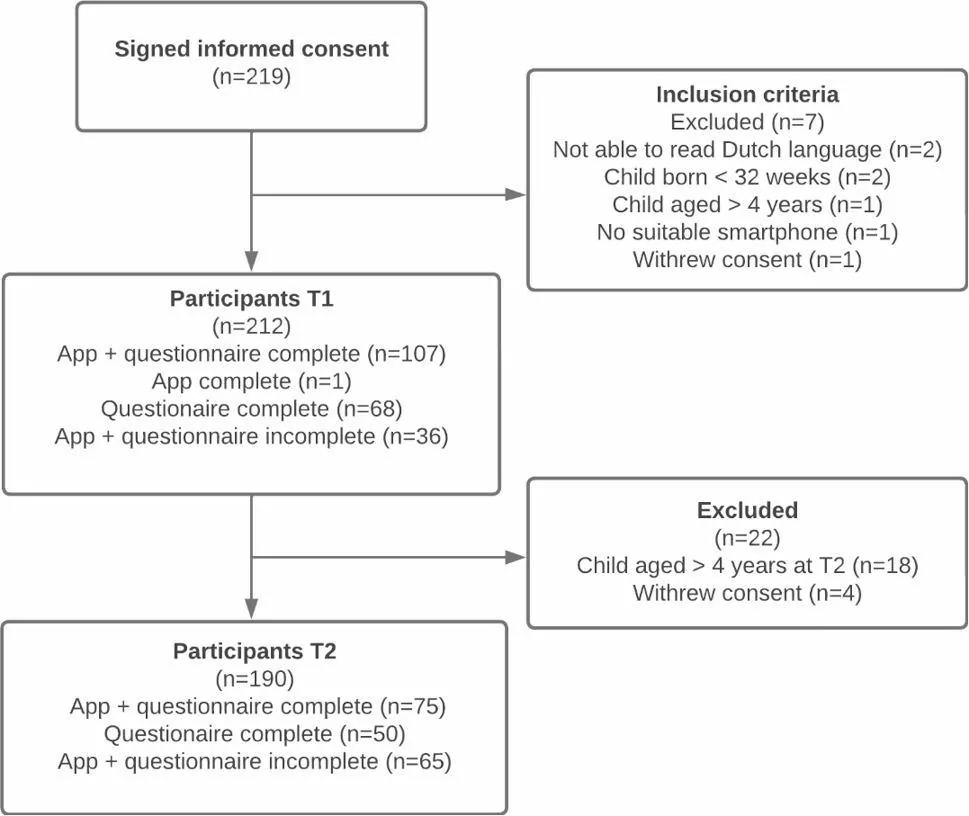
Participant flowchart quantitative sub-study

Figure 2 presents the participant flowchart of the quantitative sub-study. In total, 219 parents initially signed consent for participation, with 213 parents and children meeting the inclusion criteria. Complete data (screen time, factors influencing screen time, child’s age and sex) were available for 107 children at timepoint 1 (mean age 21.3 ± 12.1 months, 43.9% girls), and 75 children at timepoint 2 (mean age 28.1 ± 10.6 months, 37.3% girls). Nineteen children were not followed up at timepoint 2 because they had turned 4 years old. The average time between timepoint 1 and timepoint 2 was 8.6 months, ranging from 6.3 to 10.1 months. Table 4 provides an overview of the characteristics of participating children and their parents at timepoint (1) The majority of participating parents identified as female (94.3%) and were highly educated (77.6%). Median screen time per day was 5 min (IQR = 0–21) at timepoint 1, and 22.5 min (IQR = 0–40) at timepoint (2) Table 5 presents the percentages for screen time intensity, the type of screen used, and the presence of others during screen time at both timepoints. Most screen time was categorized as calm (91.1% at timepoint 1 and 91.7% at timepoint 2), screen time frequently occurred in the presence of one or more adults (41.9% at timepoint 1 and 38.5% at timepoint 2), and most often took place using a television (71.8% at timepoint 1 and 61.0% at timepoint 2). Table 5 also presents the frequencies or medians and IQRs for all factors potentially influencing children’s screen time across both timepoints.

**Table 4:** Background characteristics of participants in the quantitative study at timepoint 1 (*n* = 107)

Age child in months (mean ± SD)	21.3 ± 12.1
Sex child (girl / boy)	47 / 60
Gender parent (female / male)	101 / 6
Age parent in years (mean ± SD)	35.2 ± 3.8
Level of education parent (lower / medium / higher)	2 / 10 / 83
Country of birth mother (the Netherlands / other)	89 / 18
Country of birth father (the Netherlands / other)	91 / 16

**Table 5:** Children’s screen time characteristics, and potential influencing factors at both timepoints

Screen time	Timepoint 1	Timepoint 2
Screen time in minutes (median [IQR])	5.0 [0–21]	22.5 [0–40]
Activity intensity (%)		
Calm screen use	91.1	91.7
Active screen use	6.0	5.2
I don’t know	2.9	3.1
Others present during screen time (%)		
One or more other children	12.4	19.8
One or more other adults	41.9	38.5
Both other children and adults	28.6	35.0
Child was alone	17.1	6.6
Screen type (%)		
TV	71.8	62.0
Tablet	20.4	21.1
Smartphone	6.2	9.9
Game console or computer	1.2	4.7
Other	0.4	2.3

Parental and physical environmental factors	Timepoint 1	Timepoint 2
Promoting screen time for education (scale 0–5, median [IQR])	0.0 [0.0–1.0]	1.0 [0.0–1.0]
Role modelling (scale 0–5, median [IQR])	4.0 [3.0–5.0]	3.0 [2.0–4.0]
Promoting screen time to control or reward behavior (scale 0–5, median [IQR])	0.5 [0.0–1.0]	0.8 [0.3-1.0]
Rules around screen time (scale 0–2, median [IQR])	1.0 [0.0–2.0]	2.0 [0.0–2.0]
Monitoring of screen time (% yes)	87.9	90.5
Presence of digital devices in the living room (% yes)	86.9	84.0
Availability of active play equipment indoors (scale 0–11, median [IQR])	4.0 [4.0–5.0]	4.0 [4.0–6.0]
Temperature (°C, median [IQR])	21.0 [13.4–24.1]	14.0 [10.8–19.6]

#### Associations between examined factors and children’s screen time

Table 6 presents the associations between all examined factors and children’s screen time. An increase in child age was significantly associated with increased screen time (B = 0.84, 95% CI = (0.59, 1.09)), with a moderate effect size. Furthermore, the parenting practice of ‘Promoting screen time to control or reward behavior’ was significantly associated with increased screen time (B = 13.01, 95% CI = (7.71, 18.32)), with a moderate effect size. Additionally, a significant interaction effect was observed between children’s age and this parenting practice (B = 0.48, 95% CI = (0.13, 0.83)). Stratified analysis by age group (0–16, 17–32, and 33–48 months) showed significant associations between the parenting practice of ‘Promoting screen time to control or reward behavior’ and screen time in all age groups (0–16 months: B = 6.45, 95 CI = (2.86, 10.05); 17–32 months: B = 8.01, 95% CI = (0.61, 15.41); and 33–48 months: B = 29.67, 95% CI = (9.51, 49.83)), with the strongest association in the oldest age group.

**Table 6:** Associations between included factors and children’s screen time

Factor	Unstandardized B (95% CI)	Standardized β (95% CI)	*p*-value
Child age	0.84 (0.59, 1.09)	0.32 (0.22, 0.41)	< 0.001
Child sex	-0.94 (7.42, 5.74)	-0.03 (-0.23, 0.18)	0.776
Promoting screen time for education	2.07 (-0.83, 4.97)	0.07 (-0.03, 0.17)	0.160
Role modelling	0.43 (-1.28, 2.14)	0.02 (-0.06, 0.10)	0.620
Promoting screen time to control or reward behavior	13.01 (7.71, 18.32)	0.26 (0.16, 0.37)	< 0.001
Rules around screen time	-2.09 (-5.03, 0.85)	-0.06 (-0.15, 0.02)	0.161
Monitoring of screen time	1.09 (-6.09, 8.27)	-*	0.764
Presence of digital devices in the living room	0.20 (-8.44, 8.84)	-*	0.963
Availability of active play equipment indoors	-0.46 (-2.12, 1.20)	-0.02 (-0.10, 0.06)	0.573
Maximum temperature	-0.02 (-0.05, 0.00)	-0.05 (-0.11, 0.01)	0.103

*Not applicable for dichotomous factors

#### Comparison of qualitative and quantitative results

Table 7 presents a comparison of our qualitative and quantitative results of factors influencing screen time in young children. Except for the child’s sex, all quantitatively examined factors were reported by parents in our qualitative study as influencing children’s screen time. Both quantitative and qualitative findings indicated that increasing child age and the parenting practice of using screen time to control or reward behavior were associated with increased screen time.

**Table 7:** Comparison of qualitative and quantitative findings of factors influencing screen time

Factor	Quantitative	Qualitative
Child age	+	+
Child sex	0	0
Promoting screen time for education	0	+
Role modelling	0	+
Promoting screen time to control or reward behavior	**+**	**+**
Rules around screen time	0	−
Monitoring of screen time	0	−
Presence of digital devices in the living room	0	+
Availability of active play equipment indoors	0	−
Maximum temperature	0	−

+ indicates factors associated with increased screen time; − indicates factors associated with decreased screen time; 0 indicates no evidence supporting the factor as either increasing or decreasing screen time; Factors in bold are those supported by both qualitative and quantitative results

## Discussion

This study examined factors potentially influencing screen time in children aged 0–4 years, using a convergent parallel mixed-methods approach. In the qualitative sub-study, parents identified 46 distinct influencing factors, which were categorized into eight child-related, 19 parent-related, and 16 environmental factors. The most commonly reported factor was the use of screen time as an instrument to distract or occupy the child. In the quantitative sub-study, we found that the child’s age and the parenting practice of using screen time to control or reward the child’s behavior were significantly associated with increased screen time in young children.

Our findings reveal a consistent parental factor across both qualitative and quantitative data: the instrumental use of screens to manage children’s behavior. Similar to previous studies, we found that parents frequently allow screen use to create personal time or to complete tasks without interruption [19, 26, 51–53]. This practice has previously been described as parents’ reliance on an “electronic babysitter” [26, 53]. For example, several parents indicated relying on screen time to occupy their child(ren) when they are busy, such as with household chores, or feeling tired. Additionally, consistent with prior studies, parents indicated that they frequently use screens to regulate children’s mood and behavior, particularly to calm children [51–53]. Notably, we also found a significant interaction effect between child age and the use of screens to control or reward behavior, indicating that this parenting practice has a stronger impact among relatively older children, although the association between this practice and increased screen time was present across all age groups (i.e., 0–16, 16–32, and 32–48 months). These findings underscore the importance of addressing parents’ instrumental use of screens to manage their child’s behavior across the entire age range of 0–4 years. Although not examined in the current study, prior research also indicates that low parental self-efficacy in managing screen time is associated with higher screen use in children aged 0–4 years [53, 54]. Enhancing parents’ confidence and skills in managing children’s screen time and behavior could reduce their reliance on screens [53, 55]. For example, parents could be offered practical, age-appropriate, and concrete alternatives for activities that children can engage in independently [26, 56]. In line with this, the parents in our study identified their ability to keep children occupied with non-screen activities and children’s ability to entertain themselves with non-screen activities as factors that reduce screen time. Future studies are recommended to identify suitable non-screen activities and behavior management strategies, for example by involving parents and/or grandparents who did not frequently rely on screens during child-rearing [26].

Other practices commonly indicated by parents were the use of screens for educational purposes, setting rules, and monitoring of screen time. In contrast, we did not find significant associations for these practices in the quantitative study. However, the use of screens by parents for educational purposes aligns with findings in previous qualitative studies [22, 55, 57, 58]. Notably, a recent systematic review found that educational and interactive screen content can positively impact cognitive and language development in early childhood, while excessive and passive screen use had a negative effect [12]. These findings indicate that screen time can have beneficial effects [14]. For example, from the age of two years, television programs specifically designed for educational purposes may support early language and literacy development, as well as promote positive racial attitudes and encourage imaginative play [59]. However, the potential impact of screen time on health indicators such as myopia should be carefully considered [60]. These findings highlight the importance of balancing limiting screen time and selecting high-quality, developmentally appropriate content. Future studies are needed to further investigate how different types, durations, and content of screen time influence the health and development of young children [12]. Regarding rule setting, prior quantitative studies showed mixed results [53], though qualitative studies consistently reported that parents perceive rules as effective for reducing children’s screen time [19, 22, 57]. Both quantitative and qualitative research also underscored the importance of parental monitoring in managing children’s screen time [19, 22, 57]. Furthermore, parents identified screen use during meals as a common practice, aligning with findings in previous quantitative studies [53]. These parenting practices, such as screen use during meals, rule setting, and monitoring, represent modifiable behaviors that could be targeted to reduce young children’s screen time.

Additionally, parents indicated several parental behavioral attributes and skills. While our quantitative findings did not support an association between parental screen time and children’s screen use, this relationship has been found in prior quantitative and qualitative research [3, 17, 19, 53]. Additionally, consistent evidence from previous quantitative studies support the importance that parents place on physical activity as a factor associated with reduced screen time [17]. Regarding the influence of parental attitudes and awareness of the effects of screen time, findings from previous quantitative studies were inconsistent [17, 53], whereas qualitative studies generally emphasize the relevance of these factors [21, 26, 57, 58]. Addressing these factors, such as increasing awareness of current screen time recommendations and the negative effects of parental screen use on child development, may help reduce screen time in early childhood [3, 26].

Regarding child factors, while previous quantitative studies reported inconsistent findings regarding the association between age and screen time in young children [17], both the qualitative and quantitative data in our study identified older age as a factor of increased screen time. Interestingly, a recent longitudinal study in children aged 4 to 7 years found no significant association between age and screen time, possibly due to a smaller increase in screen time after age 4 [56]. Findings on the influence of children’s tiredness and illness suggest that parents allow more screen time when children have low energy levels, consistent with research showing a negative association between sleep duration and screen time in young children [17]. However, previous studies also show that excessive screen time can impair sleep, indicating a possible bidirectional relationship [10]. Additionally, parents noted that children’s interest in screen time varies between children. This suggests that screen time in early childhood may be influenced by individual personality traits, an association not supported by existing quantitative studies [17].

Physical environmental factors indicated by parents, such as bad weather, low availability of play equipment, and the presence of screens, are consistent with findings in few previous qualitative studies [19, 26]. However, similar to previous quantitative studies [17], these were not confirmed in our quantitative analyses. Notably, although most associations observed in our quantitative analyses were non-significant, they were all in the expected direction. It is possible that our reduced sample size limited the statistical power to detect relevant effects. Therefore, further research with larger samples is warranted to more conclusively investigate these factors. Nevertheless, based on our qualitative findings, we recommend considering these physical environmental factors when aiming to reduce young children’s screen time.

Multiple social environmental factors were identified by parents in our qualitative study. While previous quantitative studies found no significant association between the number of children in the household or birth order and screen time [17], findings from previous qualitative studies with parents emphasized that having multiple siblings can make it difficult for parents to limit screen time in younger children [19, 55]. We recommend that future studies examine these factors further.

In line with our qualitative findings on policy factors, screen time limiting policies at ECEC services in the United States have been associated with decreased screen time in preschoolers [61]. However, a recent international scoping review found that only 61% of policy documents from ECEC services included specific screen time recommendations, with most of the evaluated documents published in the United States [62]. This indicates that screen time guidelines should be more consistently incorporated into ECEC policies to better support healthy screen use among young children.

### Strengths and limitations

A key strength of our study is the use of a mixed methods design with both qualitative and quantitative methods to identify factors influencing screen time in children in the entire age range of 0–4 years. The exploration of a broad range of influencing factors is a strength of the qualitative sub-study. Additionally, the longitudinal design is a strength of the quantitative sub-study.

However, the study also has limitations. In the qualitative sub-study, several of the identified factors were overlapping or closely related (e.g., the child-related factor “mood” versus the parent-related factor “using screen time to calm the child”). This made merging factors into completely unique factors challenging and potentially subjective at times. Furthermore, the age groups in the qualitative sub-study were based on chronological age and are therefore somewhat arbitrary with respect to developmental status. This may have resulted in some overlap in the factors identified across the age groups. This may have resulted in some overlap in the factors identified across the age groups. Moreover, although the stepwise approach used in our focus group sessions facilitated the identification of many factors, our findings are primarily based on the frequency with which factors were mentioned by parents. This does not necessarily indicate what parents consider most important or relevant in reducing their child’s screen time. Therefore, we recommend that future qualitative studies examine these aspects in greater depth. In the quantitative sub-study, screen time and most influencing factors were parent-reported, introducing possible recall bias and social desirability bias [63]. Furthermore, complete data were available for only 50.4% of children at the first timepoint and 39.5% at the second timepoint, resulting in a relatively small and selective sample size. This reduced statistical power limited the number of potential factors and interactions we were able to examine. Lastly, the samples in both qualitative and quantitative studies consisted predominantly of highly educated females, which may limit the generalizability of the findings.

## Conclusions

This study identified a wide range of factors potentially influencing screen time in children aged 0–4 years, including child-related, parental, and environmental factors. A key factor confirmed in this mixed-methods approach is the instrumental use of screen time to manage children’s behavior. This parenting practice could be targeted to reduce screen time in early childhood, for example by providing parents with practical alternatives to screen use, along with effective strategies for behavior management.

## Data Availability

Data that support the findings of the study are available from the corresponding author upon reasonable request.

## Abbreviations

CI: Confidence interval
ECEC: Early childhood education and care
EPAO: Environment and Policy Assessment and Observation
IQR: Interquartile range
